# Surface Evolution and Visible-Light Photocatalytic Activity of Sol–Gel Derived Co_3_O_4_ Thin Films as a Function of Annealing Temperature

**DOI:** 10.3390/gels12040345

**Published:** 2026-04-20

**Authors:** H.I. Barragán-Méndez, Y.J. Acosta-Silva, S. Rivas, S. Gallardo-Hernández, A. Méndez-López

**Affiliations:** 1 Graduate Program, Facultad de Ingeniería, Universidad Autónoma de Querétaro, Cerro de las Campanas s/n, Las Campanas, Querétaro 76010, Mexico; 2 Cuerpo Académico de Nanotecnología y su Aplicación (UAQ-CA-160), Facultad de Ingeniería, Universidad Autónoma de Querétaro, Cerro de las Campanas s/n, Las Campanas, Querétaro 76010, Mexico; 3 Departamento de Física, Centro de Investigación y de Estudios Avanzados del Instituto Politécnico Nacional, Av. IPN 2508, Ciudad de México 07360, Mexico

**Keywords:** Co_3_O_4_ thin films, photocatalytic activity, methylene blue dye, adsorption, visible light

## Abstract

Thin-film cobalt oxides have attracted increasing attention due to their visible-light activity and potential environmental applications. In this work, Co_3_O_4_ coatings were prepared on glass substrates through a sol–gel dip-coating process followed by thermal treatment at 450, 500, and 550 °C. Structural characterization was carried out using X-ray diffraction (XRD) and Raman spectroscopy. Diffraction patterns, together with the Raman spectra, indicate the formation of the cubic spinel phase of Co_3_O_4_, while sharper diffraction peaks appeared at higher annealing temperatures, indicating improved crystallinity of the films. Surface morphology was analyzed by scanning electron microscopy (SEM) and atomic force microscopy (AFM). SEM observations revealed continuous polycrystalline coatings, whereas AFM measurements showed clear variations in surface topography and roughness produced by thermal treatment. Wettability measurements obtained from contact angle (CA) analysis indicate modifications in the surface properties of the films as the annealing temperature changes. Optical characterization performed by ultraviolet–visible spectroscopy (UV–Vis) showed strong absorption in the visible region with an indirect band gap close to 1.58 eV. Photocatalytic activity was evaluated through the degradation of methylene blue under visible-light irradiation. Degradation efficiencies of approximately 93.9%, 97.4% and 98.7% were obtained after 5 h for films annealed at 450, 500, and 550 °C, respectively.

## 1. Introduction

Metal oxide semiconductors remain central to a wide range of energy-related technologies because they combine chemical stability, low cost, and structural versatility. Within this broad family, transition metal oxides have been extensively explored as functional materials for catalysis, sensing, energy conversion, and optoelectronic devices [1,2,3,4,5]. However, not all oxide semiconductors meet the optical and electronic requirements demanded by solar-driven applications. Copper oxide (Cu_2_O), for example, has been widely investigated as an absorber layer in all-oxide photovoltaic architectures. Despite its favorable abundance and p-type conductivity, its band gap of approximately 2.2 eV limits efficient utilization of the AM1.5G solar spectrum [6,7,8]. For single-junction photovoltaic devices, absorber materials with band gaps in the range of 1.1–1.6 eV are generally required, motivating the search for alternative oxide semiconductors with more suitable optical properties [9].

In this context, cobalt oxide in its spinel structure (Co_3_O_4_) has attracted increasing attention as a p-type semiconductor. Co_3_O_4_ crystallizes in the normal spinel structure Co^2+^[Co^3+^]_2_O_4_, where Co^2+^ ions occupy tetrahedral sites and Co^3+^ ions occupy octahedral sites within a face-centered cubic oxygen lattice [10,11,12]. From an optical perspective, Co_3_O_4_ exhibits multiple electronic transitions in the visible region. Reported band gap values span a broad range, including transitions around 1.24–1.60 eV, higher-energy features between 2.0 and 2.6 eV, and additional absorptions associated with deeper electronic states [13,14,15,16,17,18]. Among these, the lower-energy transition near 1.5 eV is particularly attractive for solar-related applications, as it approaches the optimal value for efficient solar absorption. In addition, the intrinsic p-type character of Co_3_O_4_ enables its use as an absorber layer in photovoltaic and photoelectrochemical systems [19,20]. Beyond optical considerations, Co_3_O_4_ has been widely studied for its electrochemical and catalytic performance. Applications include supercapacitors, lithium-ion battery electrodes, gas sensors, oxidation and reduction catalysts, and electrochromic and spintronic devices [21,22,23]. Its relatively high chemical stability, electrical conductivity on the order of 10^−2^ S·cm^−1^, and capacity for lattice oxygen exchange with the surrounding atmosphere further support its relevance for advanced energy applications [24]. To exploit these properties, Co_3_O_4_ thin films have been fabricated using a wide range of deposition techniques. Physical and chemical methods such as sputtering [25], pulsed laser deposition [26], chemical vapor deposition [17], electrodeposition [27], spray pyrolysis [20], hydrothermal approaches [18], and solution-based routes including spin-coating [28] and dip-coating [29] have all been reported. Each preparation route provides certain benefits, but also involves drawbacks associated with processing cost, scalability, control over microstructure, or reproducibility. The sol–gel route provides a versatile platform for the synthesis of oxide thin films, allowing for precise control over the composition, homogeneity, and microstructure through gel formation and subsequent thermal treatment. In particular, the transition from a polymeric gel network to a crystalline oxide phase plays a key role in defining the final surface morphology and functional properties. This aspect is especially relevant in Co_3_O_4_ systems, where subtle variations during gel evolution and annealing directly influence catalytic behavior and light-driven activity. In this context, sol–gel approaches continue to be of interest because of their experimental simplicity, reduced processing costs, and their capability to generate homogeneous oxide films over extended areas [30].

The Co_3_O_4_ thin films fall within the nanoscale regime, where structural features strongly influence their physical and chemical behavior. In this type of material, grain size, surface states, and defect distribution play a central role, particularly when dimensions approach the nanometer range. Such conditions lead to a high density of interfaces and active sites, which are known to affect optical absorption and catalytic response. As a result, these coatings can be treated as nanostructured systems. Also, for sol–gel-derived Co_3_O_4_ thin films, the thermal treatment applied after deposition critically defines the final material response. Changes in annealing temperature affect phase composition, grain evolution, surface morphology, and both optical and electrical properties [31]. At temperatures below ~400 °C, the coexistence of CoO and Co_3_O_4_ phases is commonly reported. Upon increasing the annealing temperature, the spinel Co_3_O_4_ phase progressively dominates, together with grain growth and a measurable increase in electrical conductivity, often related to reduced grain-boundary effects [32,33]. Even so, reported optical band gap values remain widely scattered in the literature, and a direct microstructure–optical response relationship is not consistently observed, particularly for sol–gel Co_3_O_4_ thin films deposited on glass substrates and treated at moderate temperatures.

Changes in microstructure play a key role in the physical and catalytic behavior of Co_3_O_4_ thin films. Although cobalt oxide has been widely investigated, the relationship between thermal treatment and the resulting structural and surface characteristics of thin films is still not fully clarified, particularly when these parameters are connected with optical response and photocatalytic behavior. Annealing temperature strongly affects the grain growth, surface morphology, and the defect structure of the coatings. These factors are closely related to their functional performance. In this context, the present work examines Co_3_O_4_ thin films obtained by sol–gel deposition and evaluates the influence of calcination temperature on their structural, morphological, optical, and photocatalytic properties through a combined analysis using XRD, Raman spectroscopy, SEM, AFM, CA, and UV–Vis spectroscopy. This work analyzes the evolution of the thin film microstructure and its effect on photocatalytic activity under visible-light irradiation.

## 2. Results and Discussion

### 2.1. X-Ray Diffraction

**Figure 1:**
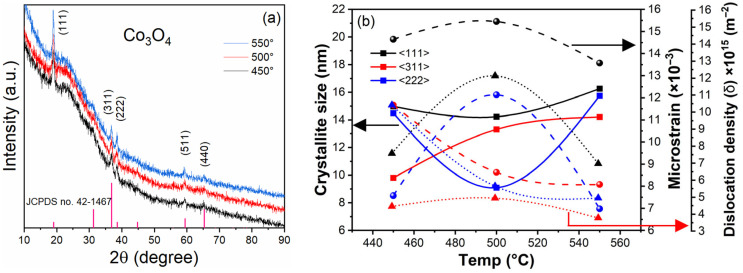
(**a**) XRD pattern of the Co_3_O_4_ films with reflections of the cubic spinel phase. (**b**) Structural parameters extracted from the XRD analysis.

X-ray diffraction (XRD) patterns of the obtained samples were recorded in the 2θ range of 10°–90° in order to evaluate the crystal structure and phase composition of the Co_3_O_4_ thin films. The diffraction pattern showed the presence of the spinel Co_3_O_4_ phase, with reflections assigned to the $\mathrm{Fd}\overset{-}{3m}$ space group, consistent with a cubic structure (a = b = c). The reflections indexed as (111), (311), (222), (511), and (440) can be observed in Figure 1a and are in agreement with the standard JCPDS card no. 42-1467, indicating the formation of the Co_3_O_4_ spinel phase. The structural parameters obtained from the diffraction data are summarized in Figure 1b. The values of crystallite size, microstrain, and dislocation density did not follow a strictly monotonic trend with annealing temperature. The film treated at 550 °C exhibited the largest crystallite size together with the lowest microstrain, which is consistent with a more defined crystalline structure. At intermediate temperature (500 °C), smaller crystallites and higher strain values were observed, indicating a higher degree of lattice distortion. This behavior can be understood considering that thermal treatment promotes both grain growth and defect rearrangement. As temperature increases, atomic diffusion facilitates grain coalescence; however, defect redistribution and local lattice distortions do not evolve linearly with temperature. As a result, intermediate states with higher strain can appear before reaching a more relaxed structure at higher temperature. Under these conditions, the film annealed at 550 °C showed a more developed crystalline structure, supported by the combination of larger crystallite size and reduced lattice strain.

Cobalt and oxygen atoms interact to create nucleation sites, which facilitate the creation of nanocrystals by means of electron transfer mechanisms. A high degree of crystallinity, which is frequently linked to an increase in crystallite size, is suggested by the strong diffraction peak seen at 19.01°. Comparable observations appear in previous studies. For example, nanostructured coatings obtained by chemical deposition were examined by Wang and co-workers, who reported XRD patterns characteristic of the spinel Co_3_O_4_ phase [34]. The observed diffraction peaks and their corresponding Miller indices were consistent with the Co_3_O_4_ phase when compared with previously reported XRD data [35,36]. After the calcination step, the cobalt hydroxide precursor was transformed into Co_3_O_4_, since no additional reflections related to secondary phases were detected. Crystallite size was evaluated from the XRD data using the Debye–Scherrer equation [37]:

(1) $$D=\frac{K\lambda }{\beta \cos\theta }$$

where K is the shape factor, typically assumed to be 0.94, and λ corresponds to the wavelength of the X-ray source used in this study (Cu-Kα, 0.15406 nm). The term β represents the full width at half maximum (FWHM) of the selected diffraction peak, expressed in radians, while θ is the Bragg angle. In this expression, D represents the crystallite size in nanometers. Table 1 lists the crystallite sizes calculated for the Co_3_O_4_ thin films. Diffraction peaks with larger width yield smaller crystallite sizes in the XRD calculations. Lattice strain and structural order in the thin films also vary under these conditions.

Oxygen vacancies modify lattice coherence and may limit crystal growth in transition-metal oxides. In Co_3_O_4_, these anion defects often lead to XRD peak broadening and smaller apparent crystallite sizes due to lattice distortion and internal stress. Lattice strain (microstrain) in the Co_3_O_4_ films was therefore examined using the Stokes–Wilson approach in order to separate the contributions of crystallite size and strain to the diffraction peak broadening [38]:

(2) $$\varepsilon =\frac{\beta }{4\tan\theta }$$

where ε denotes the microstrain, and β corresponds to the instrumental-corrected peak broadening expressed in radians. The combined use of the Scherrer and Stokes–Wilson analyses allows the contribution of crystallite size and lattice strain to XRD peak broadening to be distinguished in the Co_3_O_4_ films, providing information on the structural disorder induced by defects and thermal treatment. Xiao et al. reported that Co_3_O_4_ with a high concentration of oxygen vacancies exhibits structural modifications during reaction conditions. In their study, defect formation was associated with changes in local lattice order, which were reflected in XRD peak broadening and variations in lattice strain during electrochemical operation [39]. The dislocation density (δ) of the Co_3_O_4_ thin films prepared at different deposition temperatures was estimated from the XRD data using Equation (3) [40]. This parameter is commonly used to describe the density of lattice defects and shows an inverse dependence on the square of the average crystallite size. Thus, reduced grain size is associated with a higher defect density, which contributes to lattice strain and can influence the functional response of the films.

(3) $$\delta =\frac{1}{D^{2}}$$

Co_3_O_4_ thin films obtained by spray pyrolysis were investigated by Vijitha and co-workers, who analyzed parameters such as crystallite size, microstrain, dislocation density, and residual stress [41]. In their study, larger crystallites were linked to lower microstrain and a reduced dislocation density, whereas smaller crystallites were associated with higher defect levels. A dislocation density on the order of 1.99 × 10^15^ lines·m^−2^ was reported in that work, which is comparable to the values obtained in the present study.

The cubic lattice parameter (a) and the corresponding unit cell volume (V) of the Co_3_O_4_ nanocrystals were calculated using the geometric relationship for cubic systems (Equation (4)). In this case, the interplanar spacing d_hkl_ associated with the Miller indices (hkl) was employed to obtain the lattice constant according to:

(4) $$a=d_{hkl}\sqrt{h^{2}+k^{2}+l^{2}}$$

The unit cell volume was then calculated from the cubic relation (V = a^3^). The interplanar spacing d_hkl_ was obtained from the positions of the XRD reflections, with instrumental broadening taken into account where necessary. Small shifts in the d_hkl_ values can be associated with lattice distortions caused by defects such as oxygen vacancies, dopant incorporation, or thermal treatment. Such distortions are reflected as small changes in the lattice parameter *a* and, as a result, in the calculated unit cell volume. In the case of cubic Co_3_O_4_, Cárdenas et al. observed an increase in the lattice constant with increasing calcination temperature. A similar tendency was reflected in the unit cell volume, with the highest value reported at 550 °C (527.510 Å^3^) and the lowest at 250 °C (522.341 Å^3^) [42]. Overall, the XRD analysis suggests that thermal treatment promotes partial relaxation of internal strain and induces a slight expansion of the Co_3_O_4_ lattice. Crystallite size, microstrain, dislocation density, and lattice parameters showed measurable variations with calcination temperature in the Co_3_O_4_ films.

### 2.2. Raman Spectroscopy

**Figure 2:**
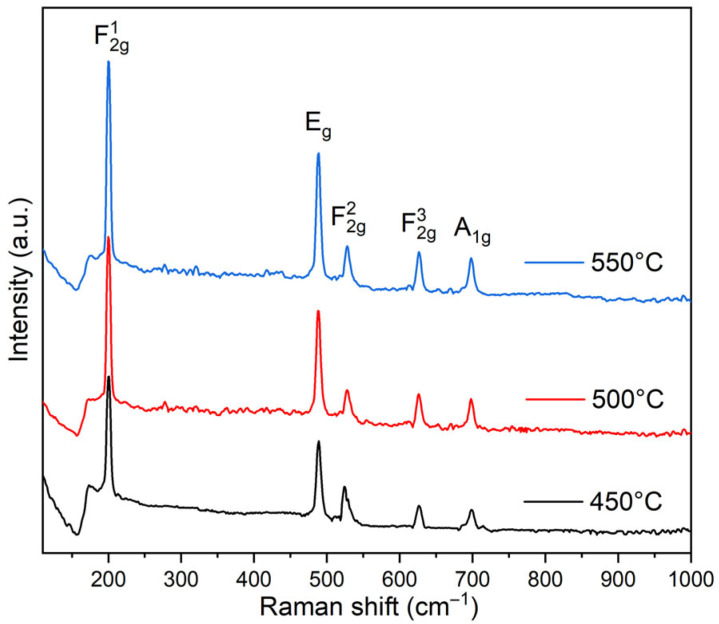
Raman spectra of Co_3_O_4_ thin films calcined at different temperatures.

Figure 2 shows the Raman spectra of the Co_3_O_4_ thin films. The spectra exhibited the five Raman-active phonon modes expected for Co_3_O_4_ with a normal spinel structure. The Raman bands were observed at approximately 198, 487, 529, 626, and 698 cm^−1^. These features are assigned to the F_2_g(1), Eg, F_2_g(2), F_2_g(3), and A_1_g vibrational modes of the Co_3_O_4_ spinel lattice, respectively. The low-frequency band near 191 cm^−1^ has been associated with F_2_g vibrations involving cobalt ions in tetrahedral coordination [43]. The A_1_g band is associated with symmetric stretching of oxygen atoms surrounding octahedral Co^3+^ sites. In contrast, the Eg and F_2_g modes arise from coupled oxygen vibrations and displacements of Co^2+^ ions in tetrahedral coordination within the spinel lattice. Similar mode assignments have been reported for Co_3_O_4_ in both single-crystal and nanostructured systems. Changes in the A_1_g band position and linewidth with thermal treatment are in line with reports on phase-pure Co_3_O_4_, where higher calcination temperatures are associated with modifications in phonon behavior [44]. In addition, Liu et al. observed an increase in the intensity of the F_2_g modes under comparable conditions, supporting the trends detected in the present films [45]. Raman spectra changed noticeably with calcination temperature. The bands became sharper and more intense at higher temperatures. XRD patterns showed the same structural tendency and no additional phases were detected. Small variations in the relative intensity and linewidth of the A_1_g and F_2_g modes were also observed. These features are often discussed in relation to local lattice disorder and the presence of oxygen vacancies in spinel oxides. The presence of these defects alters the short-range arrangement of the oxygen sublattice and affects phonon coherence. These changes are reflected in the spectral variations detected after thermal treatment.

### 2.3. UV–Vis

**Figure 3:**
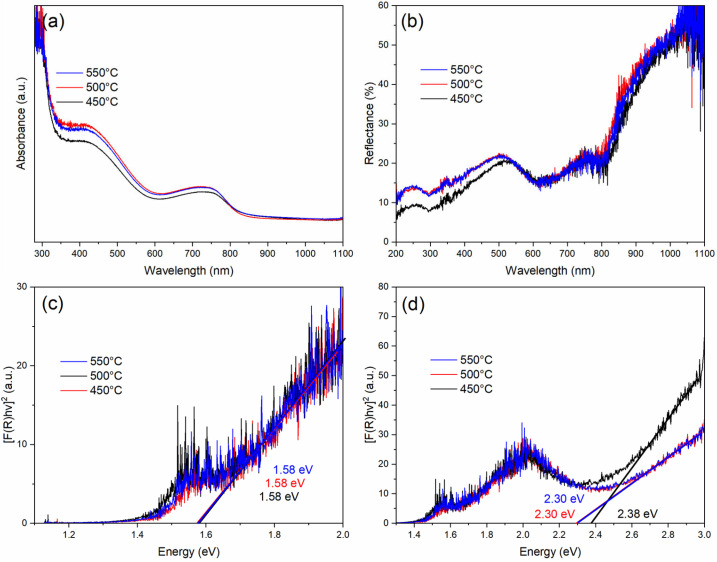
UV–Vis optical characterization of Co_3_O_4_ thin films annealed at 450, 500, and 550 °C: (**a**) absorbance spectra; (**b**) diffuse reflectance spectra; (**c**) Tauc plots used to estimate the indirect band gap (~1.58 eV); and (**d**) Tauc plots corresponding to direct optical transitions in the 2.30–2.38 eV range.

Diffuse reflectance UV–Vis measurements were carried out to examine the effect of annealing temperature on the visible-light absorption of Co_3_O_4_ thin films. Spectra corresponded to samples annealed at 450, 500, and 550 °C. The optical absorption spectra of the Co_3_O_4_ thin films appear in Figure 3a. The observed behavior can be explained by additional scattering centers and lattice defects generated during thermal treatment. Variations in grain size and surface roughness were present in the Co_3_O_4_ films and are reflected in their optical behavior. The spectra contained two main absorption regions. A first contribution was observed between 350 and 500 nm, while a wider band appeared from about 600 to 800 nm toward the near-infrared region. The absorption maximum near 434 nm has been associated with O^2−^ → Co^2+^ charge-transfer transitions. At longer wavelengths, the band observed around 750 nm is commonly related to O^2−^ → Co^3+^ transitions within the spinel lattice. Diffuse reflectance UV–Vis spectra of the Co_3_O_4_ nanocrystals recorded under ambient conditions are presented in Figure 3b. Electronic transitions in the visible region largely determine the optical response of the films, reflecting the p-type semiconducting nature commonly reported for Co_3_O_4_. The optical band gap (Eg) was determined from diffuse reflectance data through the Kubelka–Munk (K–M) method. The reflectance (R) was transformed following the relation given in Equation (5) [46]:

(5) $$F\left(R\right)=\frac{{\left(1-R\right)}^{2}}{2R}$$

Diffuse reflectance data were converted using the Kubelka–Munk function F(R), which provides an optical representation of the absorption coefficient. The band gap was determined from the corresponding Tauc plots by extrapolating the linear region of [F(R)hν]^2^ versus photon energy (hν). The fitting curves obtained from the analysis are presented in Figure 3c,d. The spectra of all films revealed two optical band gap contributions linked to charge-transfer transitions between Co^2+^ and Co^3+^ species. The lower-energy gap, around 1.58 eV, is commonly linked to an indirect transition, while a second contribution in the range of 2.30–2.38 eV corresponds to a direct transition. These values fall within the range reported for Co_3_O_4_ in both bulk and nanostructured forms and reflect the coexistence of Co^2+^ and Co^3+^ ions characteristic of the normal spinel lattice. A slight increase in the estimated band gap was observed as the crystallite size decreases. This tendency has been reported for nanocrystalline Co_3_O_4_ and is usually linked to size-related effects, where surface contributions become more relevant as the grain size is reduced [47]. The optical band gap values obtained for the Co_3_O_4_ films (1.58 and 2.30 eV) fall within the range commonly reported for undoped Co_3_O_4_ nanostructures. Warang et al. reported two direct optical transitions at 1.55 and 2.16 eV in Co_3_O_4_ films prepared by pulsed laser deposition. Their analysis related the enhanced visible-light absorption to the presence of small nanocrystalline domains, an effect commonly attributed to quantum confinement and consistent with the trend observed here [48]. Wang et al. reported nanoporous Co_3_O_4_ nanorods synthesized by a hydrothermal method, showing optical transitions at 1.28 eV and 2.34 eV. The value at 2.34 eV is very close to the band gap obtained in the present films (≈2.30 eV), suggesting a comparable optical response despite the different synthesis routes. In our case, the slightly higher absorbance may be associated with the thermal treatment applied to the films. Annealing favors improved crystallinity and the presence of surface-related defects, which introduce localized states within the band gap and contribute to enhanced visible-light absorption [49]. Taken together, the presence of two optical band gaps is consistent with the electronic structure of spinel Co_3_O_4_, where Co^2+^ and Co^3+^ ions occupy tetrahedral and octahedral sites, respectively. The agreement between the present results and previous reports obtained by the sol–gel and hydrothermal routes support the reliability of the optical response of the synthesized films. In addition, the thermal treatment plays a relevant role in modulating the band structure, mainly through changes in crystallite size and the generation of oxygen-related defects, which affect the density of localized states within the band gap.

### 2.4. Morphological Analysis

**Figure 4:**
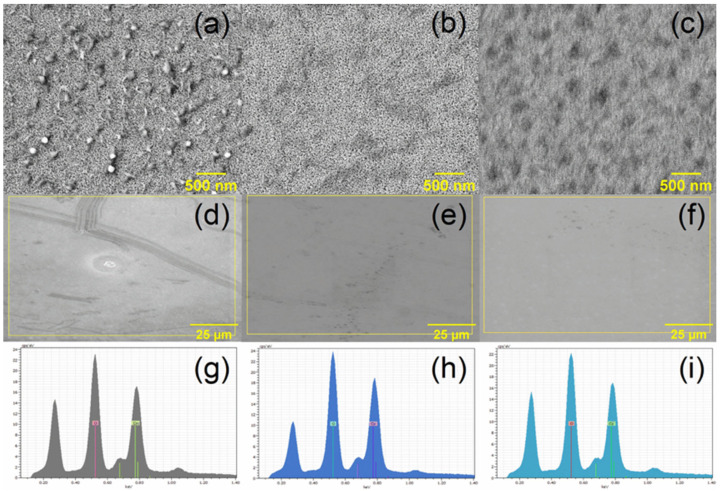
SEM images of Co_3_O_4_ thin films annealed at 450, 500, and 550 °C: (**a**–**c**) high-magnification views (×30,000); (**d**–**f**) regions selected for EDX analysis; and (**g**–**i**) corresponding EDX spectra.

SEM images of the Co_3_O_4_ thin films annealed at 450, 500, and 550 °C are presented in Figure 4. At a magnification of ×30,000, the coatings revealed agglomerated nanograins with irregular yet well-defined boundaries. An increase in grain size and pore opening was observed when the annealing temperature rose from 450 °C (Figure 4a) to 500 °C (Figure 4b) and 550 °C (Figure 4c). At higher temperatures, greater atomic mobility promotes nanograin coalescence and a partial reduction in grain boundaries, which explains the observed grain growth [50]. The film annealed at 450 °C was characterized by smaller grains and a compact surface. Larger grains, together with a more open surface morphology, were observed after thermal treatment at 500 °C and 550 °C. Comparable behavior has been observed in Co_3_O_4_ thin films subjected to thermal treatment, where higher annealing temperatures are accompanied by grain growth and surface texture changes related to grain coalescence processes [51]. EDX analysis was carried out on surface areas selected from the SEM images shown in Figure 4d–f. Analysis of the spectra obtained from these regions (Figure 4g–i) confirmed the presence of similar elements in all films. The sample annealed at 450 °C presented 40.10 wt.% O and 59.90 wt.% Co, corresponding to 71.15 at.% O and 28.85 at.% Co. Similar values were observed for the films treated at 500 °C (38.73 wt.% O, 61.27 wt.% Co; 69.95 at.% O and 30.05 at.% Co) and 550 °C (39.63 wt.% O, 60.37 wt.% Co; 70.74 at.% O and 29.26 at.% Co). The small differences among samples remained within the experimental uncertainty of the technique, indicating that the annealing temperature does not produce significant changes in the overall Co/O ratio.

### 2.5. AFM Images of Co_3_O_4_ Thin Films with Different Annealing

**Figure 5:**
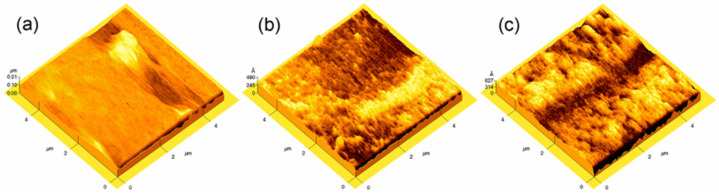
Three-dimensional AFM surface topography of Co_3_O_4_ thin films annealed at different temperatures: (**a**) 450 °C, (**b**) 500 °C, and (**c**) 550 °C.

The 3D AFM image (Figure 5) shows that the Co_3_O_4_ thin films became progressively rougher as the annealing temperature increased. At 450 °C, the surface appeared relatively smooth and continuous, with well-aggregated grains and no pronounced protrusions; the estimated roughness was very low (~2.5 nm). Grain growth became evident in the film treated at 500 °C, where the appearance of surface pores and small voids led to an increase in roughness to about 4 nm. For the sample annealed at 550 °C, the surface appeared more irregular, and the roughness reached values close to 6 nm. Changes in surface roughness appeared together with the microstructural features visible in the SEM images. Despite this change, the films still exhibited low absolute roughness values. Previous studies have report comparable behavior. Drasovean et al. obtained Co_3_O_4_ films deposited by the sol–gel method with smooth and uniform surfaces under normal atmosphere, noting that the roughness increased by approximately one order of magnitude only when the films were treated under a reducing atmosphere [52]. In contrast, Barrera et al. measured Co_3_O_4_ films deposited on metallic substrates (Cu, Fe, Al) with much higher RMS roughness values (about 42–102 nm depending on the substrate), significantly larger than those observed in this work due to differences in the deposition method [53]. The roughness values obtained in the present work (~2.5–6 nm) therefore indicate a relatively compact coating that only begins to develop surface irregularities at higher annealing temperatures, consistent with the trend observed in the AFM images.

### 2.6. Contact Angle Measurement

**Figure 6:**
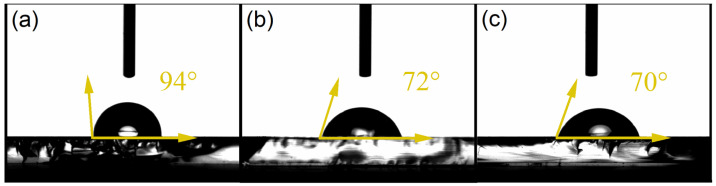
Contact angle images of Co_3_O_4_ thin films annealed at different temperatures: (**a**) 450 °C, (**b**) 500 °C, and (**c**) 550 °C.

Static water contact angle measurements were used to examine the wetting behavior of the Co_3_O_4_ thin films (Figure 6). The sample annealed at 450 °C showed a contact angle close to 94°, indicating a slightly hydrophobic surface. When the annealing temperature increased to 500 °C and 550 °C, the measured angles decreased to about 72° and 70°. These values indicate a clear shift toward a more hydrophilic surface as the annealing temperature increases. The compact structure of the film annealed at 450 °C limited the contact between the water droplet and the surface, which agrees with the higher contact angle measured for this sample. When the annealing temperature increases, the grains become larger and the surface gradually opens. Consequently, the films treated at 500 °C and 550 °C showed a rougher and more porous morphology. AFM measurements showed that the surface roughness increased as the annealing temperature rose. The films treated at higher temperatures exhibited a more irregular and porous surface, which allowed the water droplet to spread more easily across the film. In this type of oxide surface, higher roughness generally favors liquid spreading, which is consistent with the lower contact angles measured for the samples annealed at 500 °C and 550 °C. Therefore, the transition from a nearly hydrophobic surface at 450 °C to a hydrophilic behavior at 500 °C and 550 °C can be mainly attributed to morphology-driven effects, including grain enlargement, pore formation, and increased surface roughness. Similar wettability values have been reported for Co_3_O_4_ thin films prepared by different deposition techniques. For instance, Nezzari et al. reported a contact angle of about 93° for Co_3_O_4_ thin films deposited by spray pyrolysis using cobalt nitrate as the precursor and a substrate temperature of 400 °C. This value is very close to the contact angle measured for the film annealed at 450 °C in the present work (≈94°) [54]. Their study also showed that variations in precursor chemistry and surface morphology influence the wetting behavior of the films, which is consistent with the morphological effects observed in the present study. Gaikwad et al. reported hydrophilic contact angles of 63.43° and 71.68° for Co-containing oxide nanostructures, highlighting the influence of surface morphology and composition on the wetting response [55].

### 2.7. Photocatalytic Degradation

**Figure 7:**
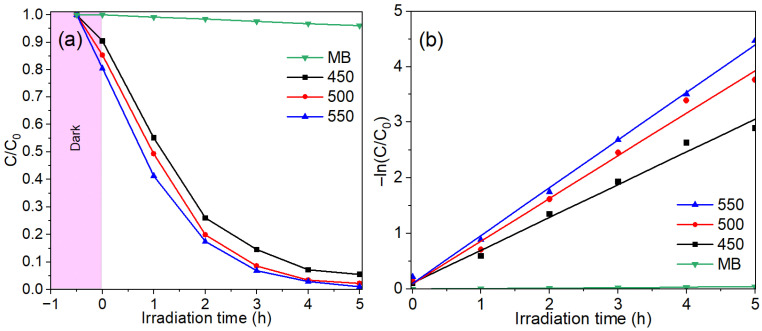
Photocatalytic degradation of methylene blue (MB) under visible light irradiation using Co_3_O_4_ thin films annealed at different temperatures. (**a**) Evolution of normalized concentration (C/C_0_) with irradiation time and (**b**) pseudo-first-order kinetic plots.

The photocatalytic activity of the Co_3_O_4_ thin films was evaluated through the degradation of methylene blue (MB) under visible light irradiation. Figure 7a shows the evolution of the normalized concentration (C/C_0_) as a function of irradiation time for films annealed at 450, 500, and 550 °C. Prior to illumination, the samples were kept in the dark to allow adsorption–desorption equilibrium between the dye molecules and the catalyst surface. A slight decrease in C/C_0_ was observed during this stage, indicating the partial adsorption of MB on the catalyst surface. Once irradiation began, a pronounced decrease in dye concentration was observed for all Co_3_O_4_ samples, whereas the MB solution without the catalyst remained nearly constant, confirming that photolysis was negligible under the experimental conditions. The degradation efficiency increased with annealing temperature. After 5 h of irradiation, the film annealed at 450 °C reached about 93.9% degradation (C/C_0_ ≈ 0.05). Higher efficiencies were obtained for the films treated at 500 and 550 °C, with final degradation values close to 97.4% and 98.7%, respectively. The improvement suggests that thermal treatment promotes a more active Co_3_O_4_ surface for the photocatalytic process. To analyze the reaction kinetics, the experimental data were fitted using the pseudo-first-order model commonly associated with the Langmuir–Hinshelwood mechanism. The linear plots of −ln(C/C_0_) as a function of irradiation time are presented in Figure 7b. The good linear correlation suggests that the degradation process follows pseudo-first-order kinetics. Furthermore, the slope of the fitted lines increases with annealing temperature, indicating a higher apparent reaction rate constant for the films treated at higher temperatures. The kinetic parameters obtained from the linear fitting confirm this trend. The apparent rate constants (k) were calculated as 0.58983 h^−1^, 0.76796 h^−1^, and 0.85932 h^−1^ for the films annealed at 450, 500, and 550 °C, respectively. The corresponding correlation coefficients (R^2^ = 0.9886, 0.98936, and 0.99761) indicate an excellent agreement with the pseudo-first-order model. The progressive increase in k suggests that thermal treatment improves the photocatalytic efficiency of the Co_3_O_4_ films. The higher photocatalytic activity observed at elevated annealing temperatures appears to be related to the structural evolution of the films. SEM images showed that the microstructure became more open and defined as the annealing temperature increased. AFM measurements also revealed a slight increase in surface roughness. These features suggest a larger number of accessible surface sites, which can favor the interaction between the catalyst surface and the dye molecules during the photocatalytic reaction.

The photocatalytic degradation of methylene blue on Co_3_O_4_ films can be described considering the generation of reactive species under visible-light irradiation. Upon light absorption, electron–hole pairs are produced, which may participate in surface redox reactions. Electrons can interact with dissolved oxygen, leading to the formation of superoxide-type species, while holes may react with adsorbed water or hydroxyl groups, giving rise to oxidizing radicals. These species are commonly reported as the main contributors to dye degradation in Co_3_O_4_-based systems [48,56].

In this work, no specific trapping experiments were carried out to identify the dominant reactive species. Therefore, the reaction pathway is presented as a general mechanism based on previous reports for similar materials. A simplified scheme is given below:

Co_3_O_4_ + hν → e^−^ + h^+^ (6)

O_2_ + e^−^ → •O_2_^−^ (7)

H_2_O + h^+^ → •OH + H^+^ (8)

MB + reactive species → degradation products(9)

The photocatalytic activity obtained in this work is in line with previous reports on Co_3_O_4_-based materials. Ganesh et al. reported the degradation of methylene blue using Co_3_O_4_ nanostructures obtained through a green synthesis route based on a tea extract [57]. Under visible light irradiation, the pristine Co_3_O_4_ sample reported in that work degraded roughly 35–45% of methylene blue within about 60–100 min. Interestingly, the degradation efficiency obtained in the present work for the film annealed at 450 °C (~39% after 1 h) was very close to the values reported for bare Co_3_O_4_ in that study, indicating comparable photocatalytic behavior despite the different synthesis routes and catalyst morphologies. A similar trend was reported by Shi et al., who studied the visible-light photocatalytic activity of Co_3_O_4_ and Co_3_O_4_@TiO_2_ core–shell structures [58]. Their results showed that pure Co_3_O_4_ achieved only about 37% degradation of methylene blue after 120 min of visible light irradiation, whereas the composite material reached significantly higher efficiencies due to improved charge separation at the heterojunction interface. In contrast, the Co_3_O_4_ thin films prepared in the present work already reached approximately 71% degradation after 120 min for the sample annealed at 450 °C, demonstrating a considerably higher activity compared with the pure Co_3_O_4_ reported in that study. Additionally, Mohandes et al. reported that biosynthesized Co_3_O_4_ nanoparticles achieved around 89% degradation of methylene blue after 105 min under UVA irradiation [59]. In the present work, the film annealed at 550 °C reached approximately 80% degradation within the first 2 h of visible light irradiation, which is comparable considering that different irradiation sources and catalyst morphologies were employed. These comparisons suggest that the Co_3_O_4_ thin films prepared in this study exhibit competitive photocatalytic activity relative to previously reported Co_3_O_4_-based systems.

Overall, the film annealed at 550 °C exhibited the highest photocatalytic efficiency, which can be attributed to the improved surface morphology, higher roughness, and enhanced availability of active sites, all of which favor the generation and participation of reactive oxygen species during the degradation process.

## 3. Conclusions

Annealing temperature strongly influences the structural evolution and photocatalytic behavior of sol–gel derived Co_3_O_4_ thin films deposited on glass substrates. Diffraction patterns and Raman spectra indicate that the films correspond to the cubic spinel phase of Co_3_O_4_, while sharper diffraction peaks observed at higher annealing temperatures reveal improved crystallinity. Surface examination showed that thermal treatment promotes grain growth and produces noticeable changes in film morphology, as observed from SEM and AFM. Contact angle measurements further indicate that the annealing process modifies the wettability of the coatings, reflecting changes in their surface properties. Optical measurements obtained from UV–Vis spectroscopy revealed strong absorption in the visible region with an indirect band gap close to 1.58 eV. Photocatalytic tests carried out with methylene blue demonstrated that degradation efficiency increased systematically with annealing temperature, reaching approximately 93.9%, 97.4%, and 98.7% after 5 h of visible-light irradiation for films treated at 450, 500, and 550 °C, respectively. The superior performance observed for the film annealed at 550 °C is attributed to the combined effect of improved crystallinity and the development of a rougher surface morphology, which promotes more efficient interfacial charge transfer and interaction with the dye solution, leading to an enhanced generation of reactive species involved in the photocatalytic process.

## 4. Experimental Details

### 4.1. Materials

Cobalt(II) nitrate hexahydrate (Sigma-Aldrich Co., St. Louis, MO, USA), ethyl alcohol absolute (J.T. Baker, purity-99.91%, Phillipsburg, NJ, USA), lactic acid, 85% (J.T. Baker, Phillipsburg, NJ, USA), and ethylene glycol (J.T. Baker, Phillipsburg, NJ, USA).

### 4.2. Substrate Cleaning

The glass substrates (Corning 2947, Corning, NY, USA) were cleaned following a procedure previously reported by Shrinatha et al. [60]. Initially, the substrates were placed in deionized water and subjected to ultrasonic agitation for several minutes in order to remove surface contaminants. After this step, the substrates were immersed in a chromic acid (H_2_CrO_4_) solution for 3 h. Finally, an additional cleaning stage was carried out by placing the substrates in a nitric acid solution at 100 °C for 1 h.

### 4.3. Preparation of Co_3_O_4_ Thin Films

A precursor solution was prepared using ethanol as the solvent. A volume of 40 mL was placed under magnetic stirring, followed by the addition of 0.005 mol of cobalt nitrate hexahydrate. The mixture was maintained at 50 °C for 10 min until complete dissolution, yielding a solution with a concentration of 0.125 mol·L^−1^. Lactic acid (0.08 mL) and ethylene glycol (0.04 mL) were then incorporated, and the solution was kept under stirring for 60 min. After this step, the solution was allowed to cool to room temperature. Thin films were deposited on previously cleaned glass substrates by dip-coating. The substrates were withdrawn from the solution at a constant rate of 2 cm·min^−1^. Twenty coating cycles were applied. After each deposition, the films were dried in air at 5 min to remove residual solvent and stabilize the layer. Once the multilayer structure was obtained, the samples were pre-treated at 250 °C for 60 min. Final thermal treatment was carried out in air at 450, 500, and 550 °C for 120 min.

### 4.4. Structural, Optical, and Morphological Characterization

The crystalline structure of the Co_3_O_4_ thin films was examined by X-ray diffraction (XRD). Measurements were carried out using a Philips PANalytical X’Pert PRO diffractometer (PANalytical B.V., Almelo, The Netherlands) equipped with Cu-Kα radiation (λ ≈ 0.154 nm). Diffraction patterns were collected over a 2θ range of approximately 20–80°, operating at 30 kV and 40 mA. Data acquisition was performed in continuous scanning mode with an angular step close to 0.02° (2θ) and an acquisition time of about 1 s per step.

Complementary structural information was obtained by Raman spectroscopy. The spectra were recorded at room temperature using a Labram Dilor system coupled to a He–Ne laser source (Dilor, Lille, France), covering the spectral region between 150 and 800 cm^−1^.

Surface features and film texture were examined by scanning electron microscopy (SEM) using a JEOL JSM-6300 instrument (JEOL Ltd., Tokyo, Japan). UV–Vis measurements were used to assess the optical response of the films, employing a Thermo Fisher Evolution 220 spectrophotometer (Thermo Fisher Scientific Inc., Waltham, MA, USA). Using an AFM, measurements were performed on the Co_3_O_4_ thin films to evaluate surface topography and roughness, using a Park Scientific Instruments system (Park Scientific Instruments, Santa Clara, CA, USA). The contact angle measurement device (Dataphysics, model OCA 50, Filderstadt, Germany) was used to assess the hydrophilic characteristics of the Co_3_O_4_ thin films.

### 4.5. Photocatalytic Activity Evaluation

A 100 W V-501 LED reflector lamp (ILV, Shanghai, China) served as the visible-light source. The device has a nominal color temperature close to 6500 K and emits mainly in the 420–780 nm spectral region. During the photocatalytic measurements, the lamp was positioned laterally at a distance of about 15 cm from the quartz cells. The irradiation intensity at the film surface was about 10 mW·cm^−2^.

For each test, the Co_3_O_4_ thin films were placed inside 3 mL quartz cells and immersed in an aqueous methylene blue (MB) solution with an initial concentration of 8.14 mg·L^−1^. Before light exposure, the system was kept in the dark to allow adsorption–desorption equilibrium between the dye molecules and the film surface. The MB concentration at the moment irradiation started was defined as C_0_ for the kinetic analysis.

At predetermined irradiation times, the lamp was temporarily switched off and individual cells were removed for analysis. Changes in MB concentration were followed by UV–Vis spectroscopy through the evolution of its characteristic absorption band over irradiation time.

A reference test performed under identical illumination conditions, in the absence of Co_3_O_4_ thin films, did not show any significant variation in MB concentration after nearly five hours of irradiation. Under these conditions, dye removal was only observed when the photocatalytic films were present.

## Figures and Tables

**Table 1:** Parameters of the prepared samples.

Calculated Parameters	450 °C	500 °C	550 °C
Lattice constant (Å)	a = b = c = 8.084	a = b = c = 8.096	a = b = c = 8.100
Unit cell volume, a^3^ (Å^3^)	528.471	530.806	531.597

## Data Availability

The original contributions presented in the study are included in the article, further inquiries can be directed to the corresponding author.
